# A Markov Chain Replacement Strategy for Surrogate Identifiers: Minimizing Re-Identification Risk While Preserving Text Reuse

**DOI:** 10.3390/electronics14193945

**Published:** 2025-10-06

**Authors:** John D. Osborne, Andrew Trotter, Tobias O’Leary, Chris Coffee, Micah D. Cochran, Luis Mansilla-Gonzalez, Akhil Nadimpalli, Alex McAnnally, Abdulateef I. Almudaifer, Jeffrey R. Curtis, Salma M. Aly, Richard E. Kennedy

**Affiliations:** 1 School of Medicine, University of Alabama at Birmingham, Birmingham, AL 35294, USA; 2 Department of Computer Science, College of Computer Science and Engineering, Taibah University, Yanbu 46421, Saudi Arabia

**Keywords:** natural language processing, personally identifiable information, data anonymization, health insurance portability and accountability act, named entity recognition, de-identification

## Abstract

“Hiding in Plain Sight” (HIPS) strategies for Personal Health Information (PHI) replace PHI with surrogate values to hinder re-identification attempts. We evaluate three different HIPS strategies for PHI replacement, a standard Consistent replacement strategy, a Random replacement strategy, and a novel Markov model strategy. We evaluate the privacy-preserving benefits and relative utility for information extraction of these strategies on both a simulated PHI distribution and real clinical corpora from two different institutions using a range of false negative error rates (FNER). The Markov strategy consistently outperformed the Consistent and Random substitution strategies on both real data and in statistical simulations. Using FNER ranging from 0.1% to 5%, PHI leakage at the document level could be reduced from 27.1% to 0.1% and from 94.2% to 57.7% with the Markov strategy versus the standard Consistent substitution strategy, at 0.1% and 0.5% FNER, respectively. Additionally, we assessed the generated corpora containing synthetic PHI for reuse using a variety of information extraction methods. Results indicate that modern deep learning methods have similar performance on all strategies, but older machine learning techniques can suffer from the change in context. Overall, a Markov surrogate generation strategy substantially reduces the chance of inadvertent PHI release.

## 1. Introduction

The removal of Personal Health Information (PHI) from medical records to prevent patient re-identification is driven by privacy considerations, legal mandates or other ethical imperatives. Synthetic text may be useful in some circumstances [1–3], but real text typically serves as the ground truth for evaluation even when synthetic text is used for training. The removal of PHI in the healthcare context is termed “de-identification”. In the United States, the governing legal framework for privacy in healthcare is the Health Insurance Portability and Accountability Act (HIPAA), which under section 164.512 of the Privacy Rule provides both the standard and implementation guidelines for two methods of de-identification. The “safe harbor” de-identification method specifies which data elements to remove. Although these elements may be targeted by de-identification software [4], this software may not replace identified PHI with realistic surrogate text. For instance, redacted text may be replaced by the category label of the PHI removed and not resemble the original text, potentially making it more difficult to train machine learning algorithms where training and test data should be similar. The challenge of creating high quality de-identified synthetic text for use with machine learning may lead even large, multi-national corporations to skip the process entirely, leading to PHI data leaks [5]. Most patient re-identification occurs when data is not de-identified according to existing standards [6].

### 1.1. Replacement Text in Existing Software

Software has been developed to de-identify clinical text; however, cost, availability, and licensing restrictions have limited public evaluations to only a subset of software [7,8]. Software evaluated has included Amazon’s Comprehend Medical [9], CliniDeID [10,11], National Library of Medicine’s Scrubber [12], NeuroNER [4] and MITRE Identification Scrubber Toolkit [13,14], Massachusetts Institute of Technology de-identification software [15] and Emory Health Information DE-identification (HIDE) software [16]. However, most de-identification software does not provide the option to output realistic replacement text, which is unfortunate because PHI identification at detection time could suggest a more appropriate replacement. Some exceptions to this include CliniDeID, Stanford’s HIPS [17] and nference De-Id [18]. CliniDeID uses entity-specific randomly selected values that are applied consistently across the record as surrogates, HIPS [17] uses curated lists to generate realistic replacements and nference De-Id [18] uses a more complex strategy including applying “entity-specific rules and heuristics to improve the fidelity of the surrogate” including pronoun and ethnicity adjustments. A summary of this software is shown in Table 1.

### 1.2. Single Occurrence Re-Identification Vulnerability in Existing HIPS Replacement Methods

A recent systematic review [22] of different de-identification methods indicates that contemporary machine learning and hybrid approaches can yield binary token-level F1-scores of over 98%. Although this number may appear high at first glance, the high occurrence of PHI in a sufficiently realistic and large corpus will still generate false negatives for HIPAA-covered entities, thereby leaking PHI. Realistic surrogate text generation strategies can address the “residual PHI problem” [23] by limiting the ability of human readers to identify approximately 70% of PHI in clinical text [24]. This “hiding-in-plain sight” (HIPS) [13] strategy is based on similarity between the original text and the synthetic replacement, making the leaked PHI inconspicuous. Thus, a HIPS approach is preferable from a re-identification perspective versus simply replacing PHI with the detected entity type, a common default output of PHI Named Entity Recognition (NER) software and used in the widely distributed MIMIC corpus. However, existing HIPS implementations consistently replace the PHI so that the patient or provider name is always replaced by the same surrogate value. The advantage of this consistent replacement strategy is the provisioning of more realistic text, the disadvantage is that the presence of a single critical missing personal identifier (such as a patient name) gives an attacker the ability to identify the patient, even without complex statistical analysis. Thus, a HIPS corpus utilizing a consistent strategy of surrogate replacement has the same “Single Occurrence Re-identification Vulnerability” (SORV) as PHI replaced by entity names. This vulnerability could expose two-thirds of the leaked PHI in an attack utilizing machine learning; therefore, further improvements to ensure privacy protection are warranted [24] given the increasing prevalence of machine learning. Another machine learning approach that HIPS is vulnerable to are “parrot attacks”, where an attacker employs a similar annotation and de-identification method on the target text as the de-identification corpus provider. The attacker then examines any false negatives to infer that PHI has been missed [25]. A HIPS approach that removed the SORV would be preferable.

### 1.3. Need for Improved HIPS Strategies

The increasing power of Large Language Models (LLMs) can render traditional HIPS strategies (“Consistent” substitution) less effective for PHI leakage. Using a similar strategy to ours, Patsakis and Lykousas [26] used the Faker library to perform a synthetic substitution of PHI to use surrogates for 11 identifiers for short snippets of text describing 20 famous celebrities. Each celebrity had 46 to 61 descriptions, with a mean count of 54 descriptions. Initially, GPT-3 [27] could identify 72.6% of celebrity descriptions, but none when celebrities were de-identified using a “Consistent” strategy. However, simple prompt engineering allowed for the identification of 69.44% of the descriptions, indicating that a “Consistent” HIPs strategy is not sufficient for the protection of PII/PHI when using LLMs. Recent advances in LLM are likely to exacerbate this situation.

### 1.4. Contribution: Evaluation of Novel HIPS Strategies

In this paper, we implement and evaluate novel HIPS surrogate replacement strategies in which PHI mentions are replaced by surrogate text without the “traditional” constraint that such a replacement must be consistent with previous replacements of the same type. For example, each mention of a patient name in a document could be replaced by a randomly selected name in our “*Random*” strategy or by a name selected from a Markov chain in our “*Markov*” strategy. We implement these two strategies and statistically quantify their privacy preserving benefits relative to the traditional “*Consistent*” surrogate replacement strategy. To our knowledge, no statistical evaluation has been done on these PHI substitution strategies using either synthetic or realistic clinical data sets and we provide the first such implementation. Finally, we quantify the impact of these substitution strategies on selected information extraction tasks on three different data sets, to determine the extent to which these strategies may impair the utility of the text for information extraction tasks since. This is because although the effect of surrogates is believed to be minimal “where identifiers are not crucial”, it is otherwise believed to have “a great impact on data utility” [28], although this has not been explored.

## 2. Materials and Methods

### 2.1. Software Implementation

**Figure 1.**
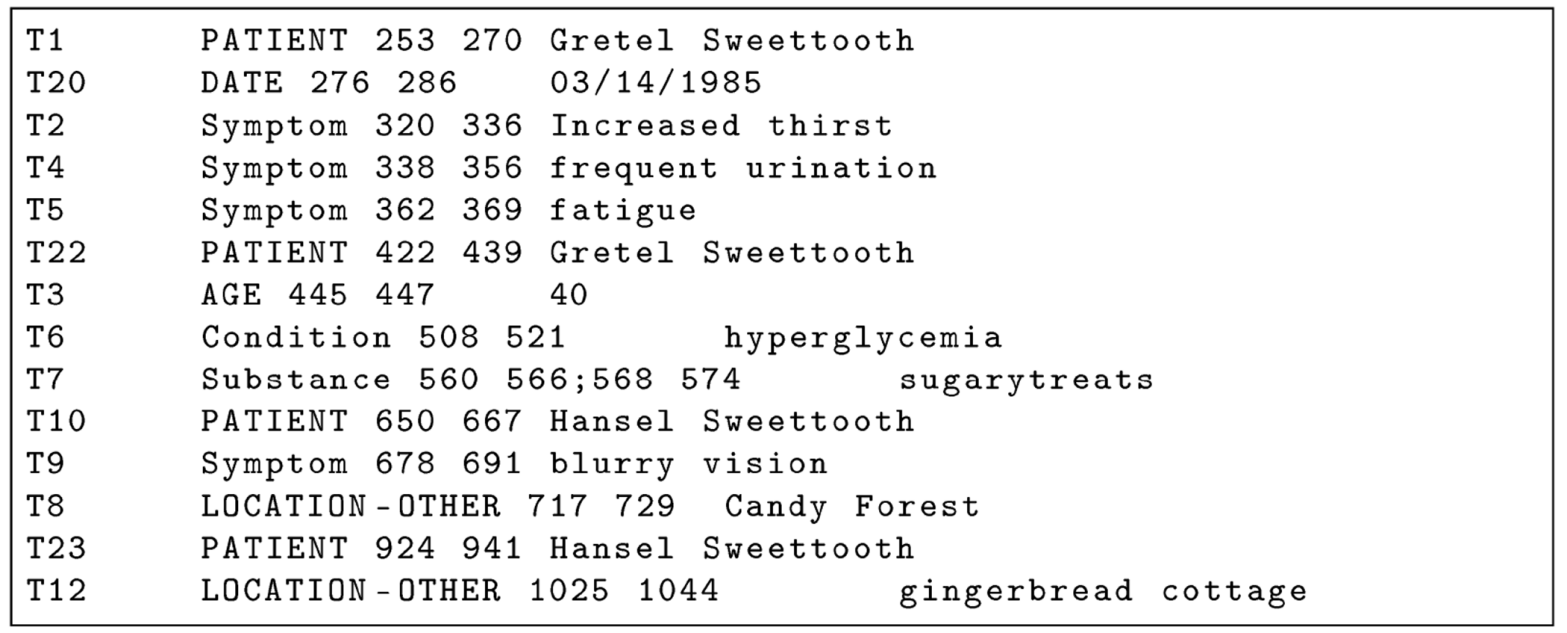
BRAT Annotation .ann file corresponding to Figure 2 clinical note. PHI annotations are capitalized.

**Figure 2.**
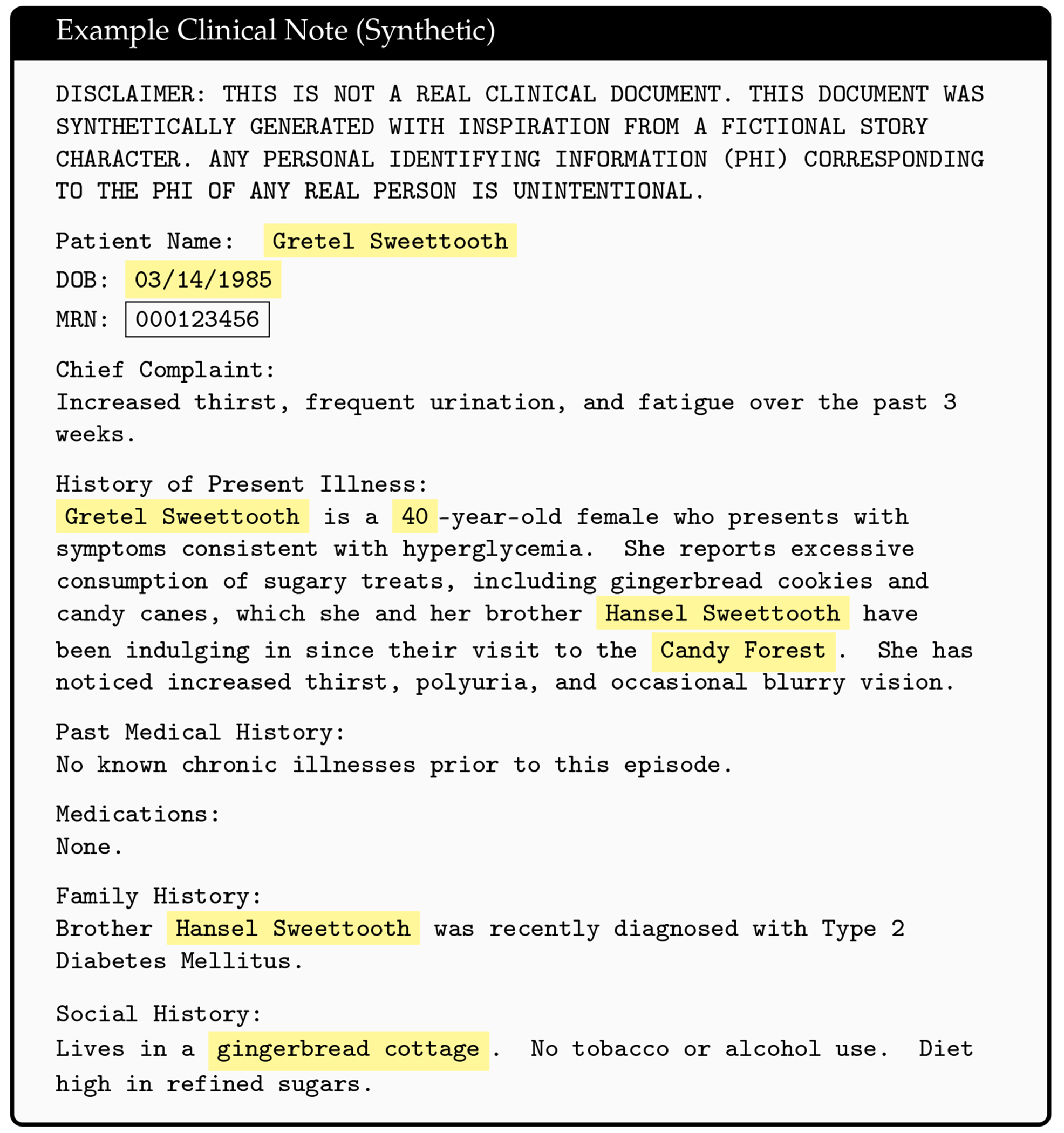
Example synthetic clinical note with identified PHI highlighted in yellow. PHI leakage results from false negatives in the de-identification software, an example of which is the MRN highlighted in the black box. Hansel and Gretel fairytale inspired synthetic clinical note was generated using Microsoft Copilot.

**Figure 3.**
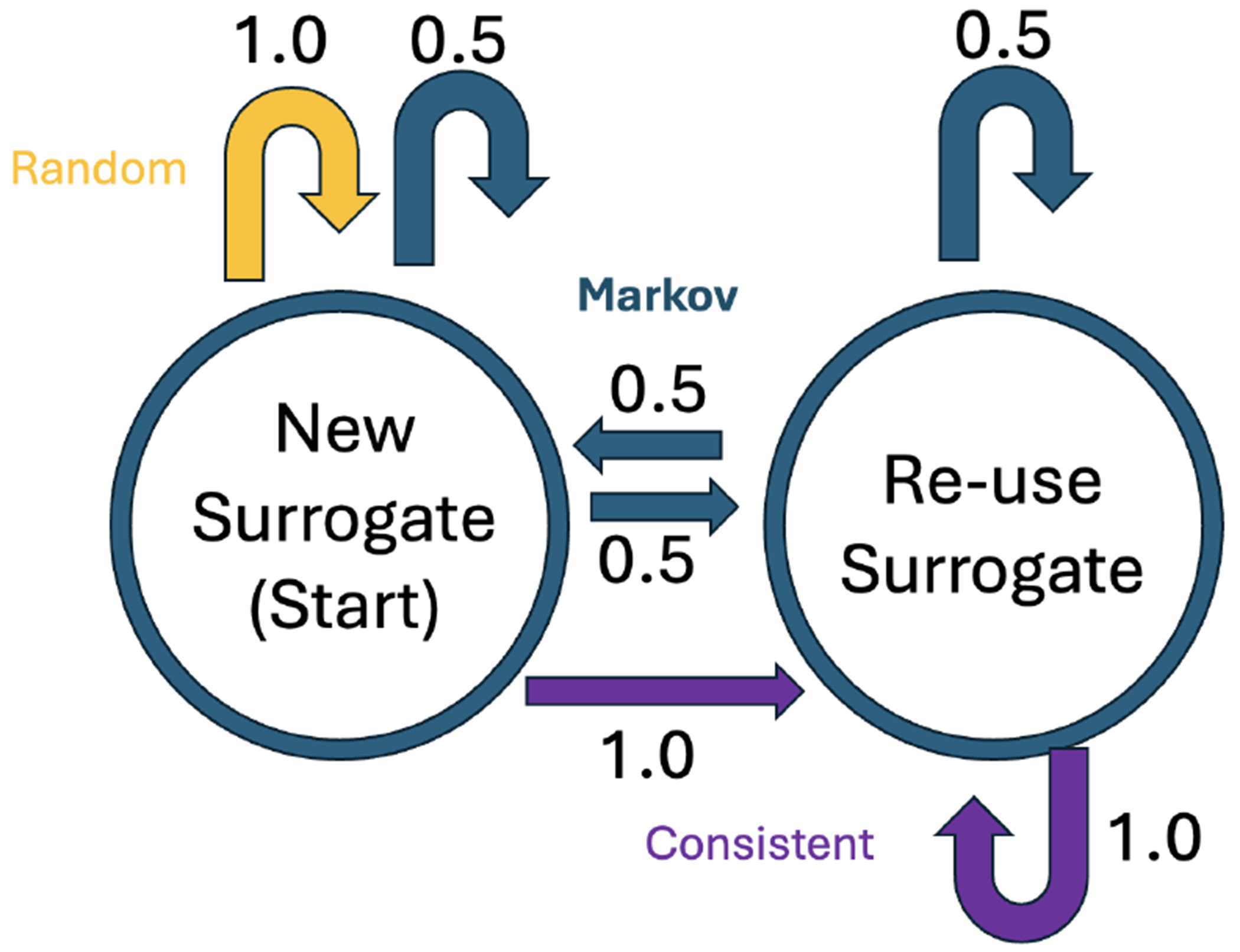
State transition diagram for HIPS replacement strategies.

We make our tool (version 0.4) and source code publicly available at https://github.com/uabnlp/BRATsynthetic (accessed on 6 September 2025). BRATsynthetic uses the BRAT [29] annotation format and the associated text file (if available) to create surrogate PHI independent of the software originally used to identify that PHI. BRATsynthetic generates realistic text for 27 categories of PHI as described in the Section 2.2.1 below. Dates and ages are offset by a random number. PHI categories are stored as entities (“T” labels) in BRAT files; associated, non-PHI annotations (attributes “A” and events “E”) may also be stored in these files, and BRATsynthetic properly maintains mapping of non-PHI annotations to PHI categories after synthetic replacements. Prior to replacement, we normalize line endings, require paired .txt/.ann files (example Figures 1 and 2), restrict substitutions to the PHI tag set, and update spans in reverse-offset order to preserve alignment. Synthetic values are produced with Faker 13.7 using a defined seed from the configuration, with format and case-preserving rules for IDs, names, and codes. Each PHI category uses a custom Maker class with unique rules for handling regular expression patterns, edge cases, and Faker function calls. Within-document mention repetition follows a two-state first-order Markov policy (reuse vs. resample) as shown in Figure 3. Automated and manual verification includes counting the number of entities, events, and attributes before and after synthetic replacement, and ensuring spans correctly align for non-PHI annotations with their corresponding synthetically replaced PHI categories. The header text, “PERSONAL IDENTIFYING INFORMATION (PHI) IN THIS DOCUMENT HAS BEEN REPLACED WITH SYNTHETIC TEXT. ANY CORRESPONDENCE TO THE PHI OF ANY REAL PERSON IS UNINTENTIONAL” is prefixed to the start of the file to reduce the risk of potential legal issues associated with the unintentional match to real persons that can occur in all HIPS strategies.

### 2.2. Evaluation Corpora

To assess the impact of surrogate substitution strategy on privacy protection, we evaluated BRATsynthetic on an in-house corpus of de-identified clinical text from 165 patients collected under IRB Protocol #300002331 (NIH Grant R01AG060993-03S1, Automating Delirium Identification and Risk Prediction in Electronic Health Records). Those notes were de-identified under IRB Protocol #300002212 “U-BRITE Deidentified Translational Data Repository for Research and Education” at the University of Alabama at Birmingham that we previously used [30] to generate .ann files as input for BRATsynthetic. The corpus includes all available EHR notes for those patients, specifically 3617 documents including 1,489,362 critical entities from both inpatient and outpatient encounters from 2014 to 2021. The UAB Discharge corpus is a subset of the main UAB Corpus containing only discharge summaries. The MIMIC Corpus is a set of MIMIC discharge summaries that was obtained as part of participation in SemEval 2014 Task 7 “Analysis of Clinical Text” [31] and are sourced from outside UAB [32]. The Opioid Use Disorder (OUD) Corpus [33] is a UAB-derived data set consisting of 3295 clinical notes from 59 patients (23 controls) from physician case referrals between 2016 and 2021. These data sets contain human-annotated BRAT files and are used to evaluate changes in the information extraction performance of the different HIPS strategies, specifically if a particular strategy uniquely affects document structure and downstream language model evaluation tasks.

#### 2.2.1. Resynthesis Elements

BRATsynthetic generates a superset of i2b2 2014 personal health information entity types [34], including standard HIPAA Safe Harbor Categories, as well as more specific category types under the BRATsynthetic “Replacement Type” column in Table 2. There is a many-to-many correspondence, where some data elements map to multiple BRATsynthetic types (ex. NAMES), others are a one-to-one mapping (ex. EMAIL), and others condense different Safe Harbor Elements into a single BRATsynthetic type (ex. IDNUM). Table 3 summarizes this and other label differences between Safe Harbor Elements and BRATsynthetic categories. BRATsynthetic does provide additional resynthesis capacity by the inclusion of the PROFESSION category (shared with I2B2) and the TIME category (not required either under HIPAA Safe Harbor or used by the I2B2), which we include due to the increasing availability of second and sub-second data collected from personal devices not anticipated in the original guidelines. BRATsynthetic currently lacks UNIQUE text resynthesis ability, where UNIQUE corresponds to a section of text that could potentially reveal personal identity such as “governor’s wife” or details of unusual accidents.

#### 2.2.2. Surrogate Substitution Strategies

BRATsynthetic implements three HIPS substitution strategies: *Consistent*, *Random*, and *Markov* as well as a baseline “*Simple*” non-HIPS strategy, where identified PHI entities are replaced by the entity name. An example substitution is shown in Table 4. All three HIPS strategies are implemented as a Markov chain with two states, one that creates a new surrogate value and another that repeats the previous surrogate value. The traditional *Consistent* strategy can be considered a simple, degenerate Markov chain with self-transition probability of 1.0. The initial state always creates a new surrogate value, but the subsequent transitions vary between strategies, as shown in Figure 3. The *Consistent* strategy always maintains the previous surrogate value, the *Markov* strategy selects a new surrogate half the time, and the *Random* strategy always selects a new surrogate. The surrogate values are selected at random from the faker [35] library for each occurrence of each type of entity.

#### 2.2.3. Maximum Surrogate Repeat Size (MSRS)

The “Single Occurrence Re-identification Vulnerability” (SORV) occurs with the *Consistent* strategy of surrogate generation because the use of the same replacement value means that a single occurrence of a false negative (FN) will be identifiable as PHI. However, should an FN occur once in the de-identified document for that entity type (leaving in the patient’s real name for example), it may not be immediately obvious with the *Random* strategy that the real name was not just another surrogate value. This is true when the number of FN errors of that critical type is lower than the maximum number of times the same surrogate value is repeated when more than one surrogate value is used. We refer to this latter value as the *“Maximum Surrogate Repeat Size”* (*MSRS*). The *Markov* substitution strategy operates in the same way as the *Random* strategy, but previously used surrogate values are much more likely to occur due to the 0.5 state transition probability. This generates a larger maximum number of times a surrogate value is repeated (larger MSRS) for each critical entity so it can better “mask” FN errors. A sample run of these strategies is shown on an entity note with six names in Table 4. BRATsynthetic applies substitutions at the document level, a patient level replacement strategy operating over multiple documents is also desirable, but not currently implemented.

### 2.3. PHI Leakage Evaluation on Real Corpora

We evaluate the protective ability of surrogate addition on the UAB Corpus, UAB Discharge Corpus, and the MIMIC Corpus by focusing on *“critical”* PHI as specified in Table 2 under the “Critical PHI” column. Table 5 provides corpus statistics on the critical entity distributions of these corpora. We define an entity as “critical” if a single FN for that entity (MRN is one such example) is sufficient to identify a patient and require immediate legal disclosure of the breach. An FN by de-identification software or humans in this critical category of PHI may be commonly identified even in a haphazard fashion by a casual reader of the document when the document employs no surrogate substitution (like MIMIC) or uses substituted text as shown in Table 4 under the *Consistent* strategy. However, a single FN would not be obvious to a reader if a *Random* or *Markov* strategy is used, since the real name would not constitute the largest minority surrogate class. Thus, the ability to detect the real name as shown in Table 4 is dependent on the overall FNER, the distribution of critical entities and FN errors within those entities and the substitution strategy used. Using our UAB and MIMIC Corpora, we simulate and assess three different resynthesis strategies for surrogate information: “*Consistent*”, “*Random*”, and “*Markov*” under four different FNER (0.1%, 0.5%, 1%, and 5%). Leakage rates are computed at the document level. A leak is considered to occur if the PHI signal (measured by the critical PHI FN count) exceeds the injected noise as measured by *MSRS*. In essence, MSRS represents the maximum frequency with which a given surrogate can repeat within a document; when the number of false negatives does not exceed this number, any leaked PHI is statistically indistinguishable from synthetic surrogates. In practice, this means that for the *Consistent* strategy where the same surrogate is repeated, any FN in a critical entity constitutes a document leak, whereas for the *Random*, and *Markov* strategies this only occurs when FN errors exceed the MSRS. A leak at the patient level as one or more document leaks. Since results can vary depending on the distribution of FN errors in a corpus, we average our results over 1000 FN error generation simulations using different random seeds. Our leakage calculations and simulations are available at https://github.com/uabnlp/BRATsynthetic (accessed on 6 September 2025) [21] and were performed using Python version 3.9.

### 2.4. PHI Leakage Evaluation on Simulated Corpora

We calculated the expected leakage probability of critical information over a range of corpus sizes, ranging from 10 to 10,000 documents. For this calculation, we considered a leak to occur if the expected number of unremoved (false negative) PHI entities in the corpus was greater than a set threshold for each of the substitution methods. For *Consistent* substitution, the threshold was set at 0, since even a single instance of a real patient entity can be readily distinguished from the single fake entity. For *Random* and *Markov* substitution, the threshold was set as the expected number of fake entities [36] based on a pool of 1000 fake entities from which to randomly select using the transition probabilities described above. The threshold for a *Random* substitution was calculated as 1.015 and for a *Markov* substitution it was 2.028. We allowed the number of entities per document to assume the values of 15, 150, and 1500, and allowed the FNER to assume the values of 1% and 5%. We then calculated the expected number of real patient entities in the corpus as the product of the FNER times the number of entities per document times the number of documents. We used the binomial distribution to determine the *p*-value of whether the expected number of real patient entities exceeded the threshold. Calculations were performed using R v4.1.2.

### 2.5. Assessment of HIPS Strategy on Information Extraction Efficacy

As shown in Table 4, the *Random* and *Markov* strategies generate text that differs from the original text or a *Consistent* HIPS strategy. This can potentially cause problems for Natural Language Processing (NLP) algorithms seeking to extract information from corpora generated using these substitution strategies. To assess the impact of this, we compare the relative performance of the different strategies on a variety of information extraction NLP tasks using different generations of NLP software. This includes the popular MedSpacy [37] software, a third-party evaluation by TriNetx that utilized a Support Vector Machine (SVM) [38] based genomic marker pipeline from Averbis and a BioBERT [39]-based encoder models for Named Entity Recognition (NER) and clinical modifiers from our prior work [40]. While it is not feasible to test all NLP tasks, the clinical modifier pipeline was selected as likely to be particularly susceptible to PHI context, especially for the “Subject” modifier, which evaluates whether an extracted Named Entity is associated with the patient or someone else, a task made more challenging when the surrogate name for the patient may change in the same document. For the MedSpacy [37] evaluation, we used the “en_core_sci_sm” model on the OUD corpus with default parameters for all tasks but “Spans”, which utilizes MedSpacy custom “TargetRules” to identify OUD entities with regular expressions. Sentences were segmented with PyRuSH, a python implementation of RuSH [41]. We computed Jenson–Shannon divergence [42] to compare distributions. The Averbis genomic marker pipeline utilized by TriNetX, first tokenizes the input text, then performs dictionary lookup for mention finding, disambiguates with a linear Support Vector Machine (SVM) that considers the context, applies rules for specific patterns, and finally performs the final classification with a linear SVM. Evaluation on the genomic marker pipeline was done on a set of 144 documents, and the results are reported and errors analyzed only for the five documents where results differed. For the BERT-based evaluation, we replicated our previous work [40], but replaced PHI using *Simple* replacement or one of the three HIPS strategies. We used Shapiro–Wilks tests to examine the normality of the mean F1 scores, with *p* > 0.05 indicating that the scores for *Consistent*, *Markov*, *Random*, and *Simple* are from the same normal distribution and are not significantly different from results for the original text.

### 2.6. BRATsynthetic Runtime Experiment

Runtime performance was evaluated on a superset of the UAB corpus consisting of 28,547 documents that have been de-identified using a BERT [43] based de-identification tool [44], but have not been manually reviewed. Corpus statistics and experimental parameters are in Table 6.

## 3. Results

### 3.1. Surrogate Replacement Strategy: PHI Leakage Assessment

**Figure 4.**
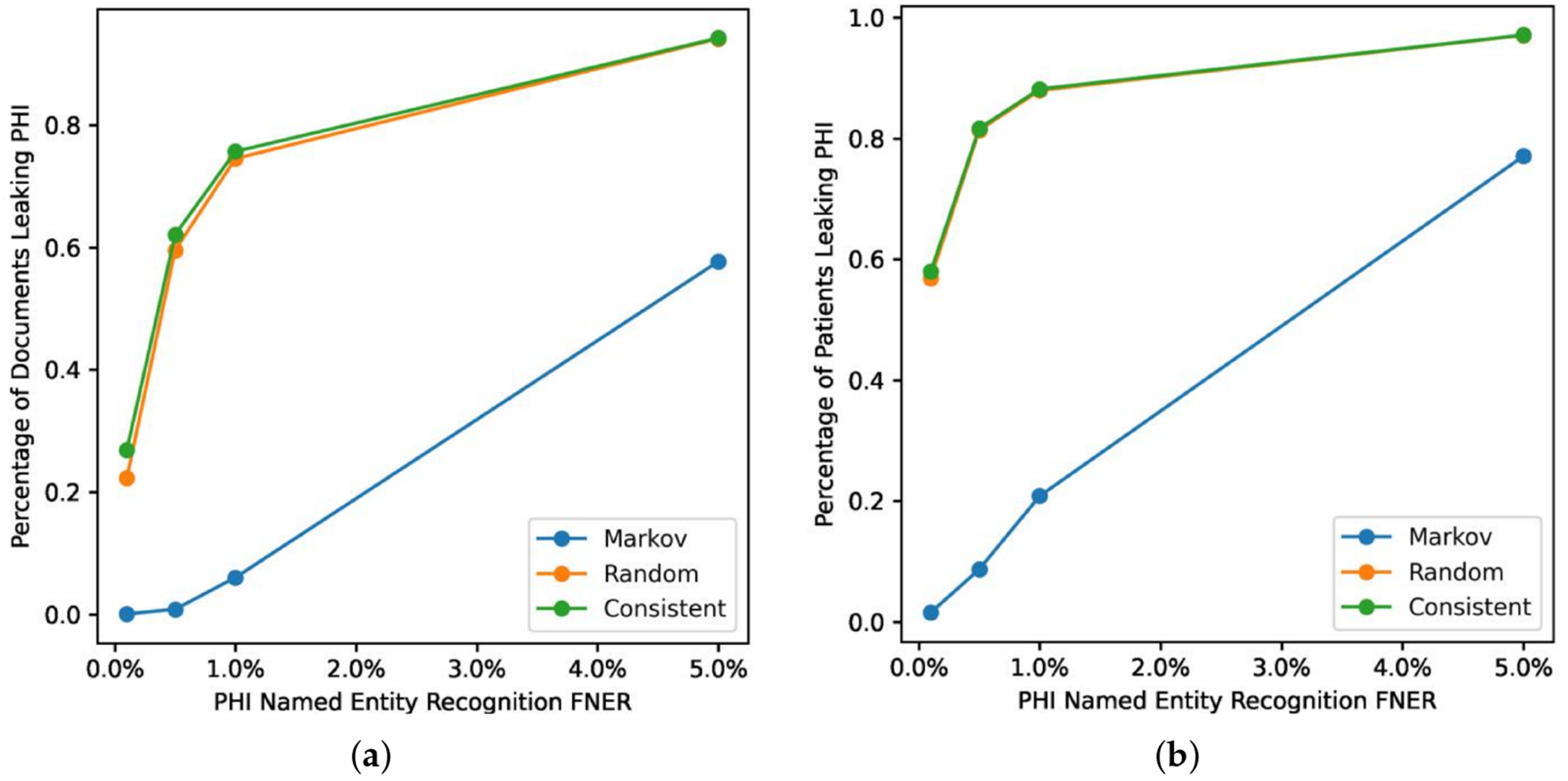
Impact of surrogate substitution strategies on PHI leakage on UAB corpus. This shows the computed leakage rate for (**a**) document level and (**b**) patient level.

**Figure 5.**
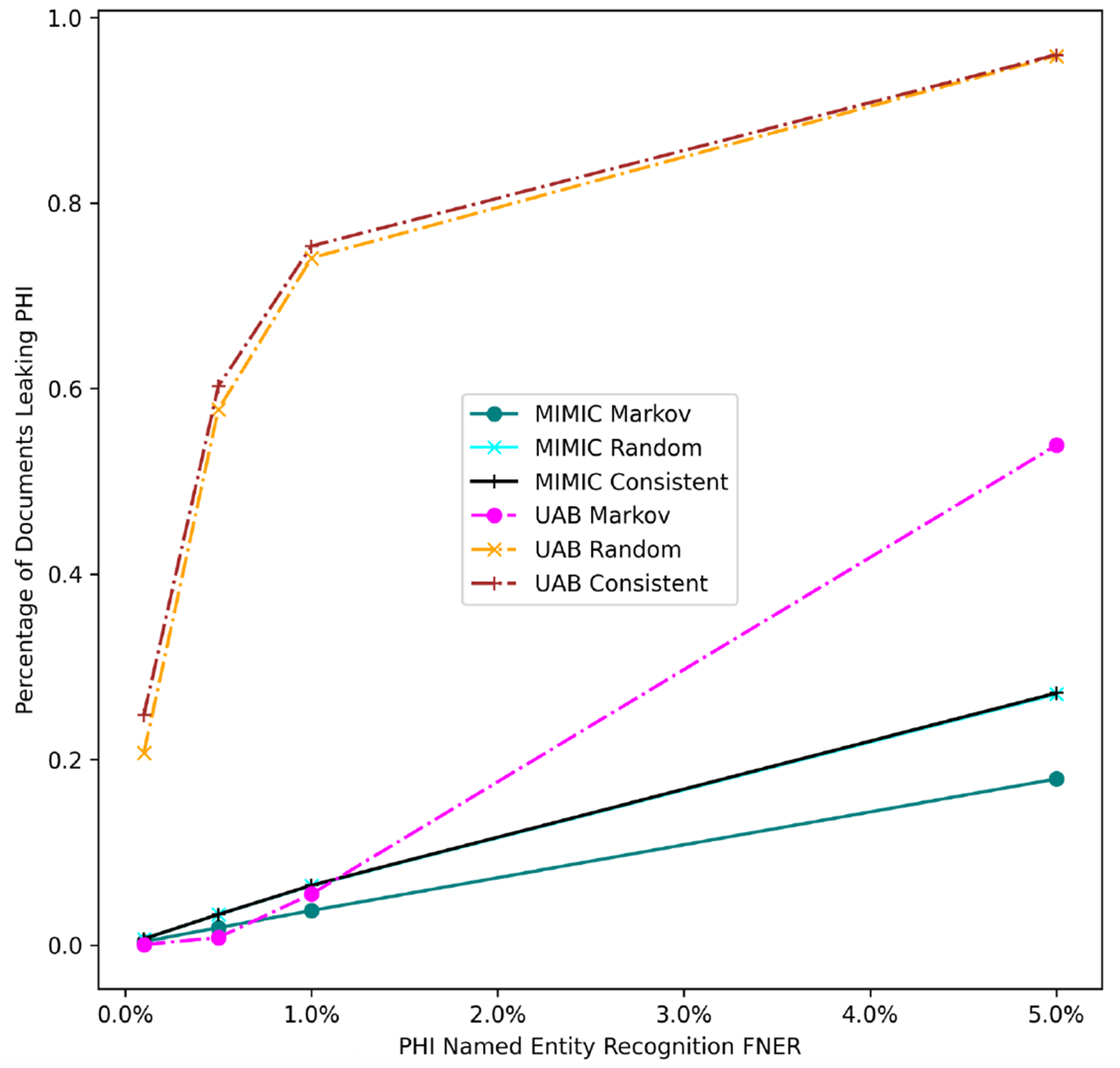
UAB and MIMIC corpus PHI document leakage. FNER is the false negative error rate.

**Figure 6.**
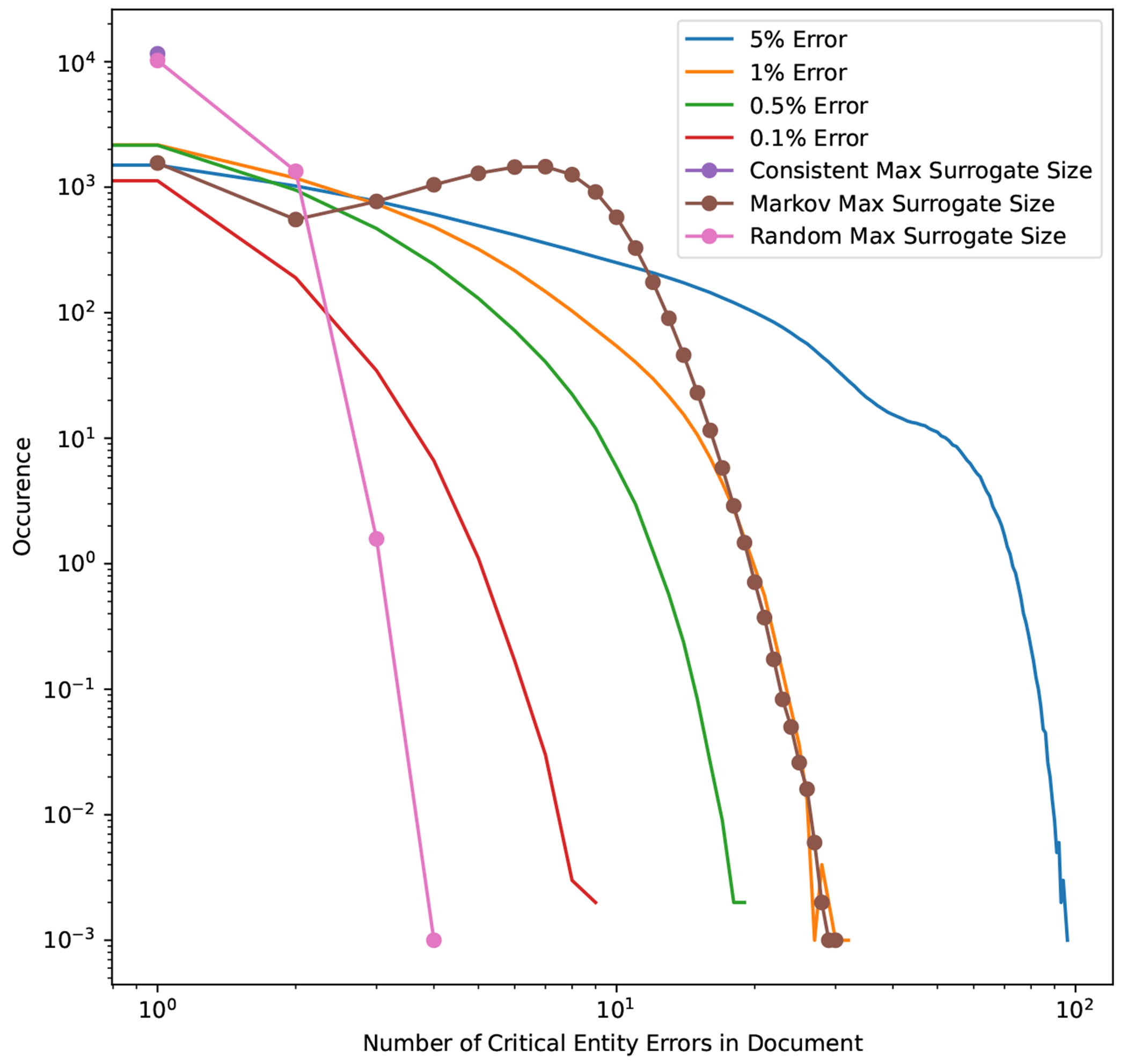
Model error masking simulation on UAB corpus. The distribution of errors across 1000 simulations, superimposed over the distribution of the MSRS for all three HIPS substitution strategies. *Consistent* substitution always reapplies the same surrogate replacement value for each class of critical PHI, so any false negatives are detectable. The *Random* substitution strategy allows for same surrogate replacement, but since the critical entity surrogate pool size is set at 1000 almost all entities use a unique surrogate. Thus, for *Random* the *MSRS* is usually 1, but it some cases it ranges from 2 to 4. The *Markov* strategy utilizes a state transition probability of 0.5 and so will generate a higher *MSRS* and generally “cover” error rates of 1% or less in our data set.

**Figure 7.**
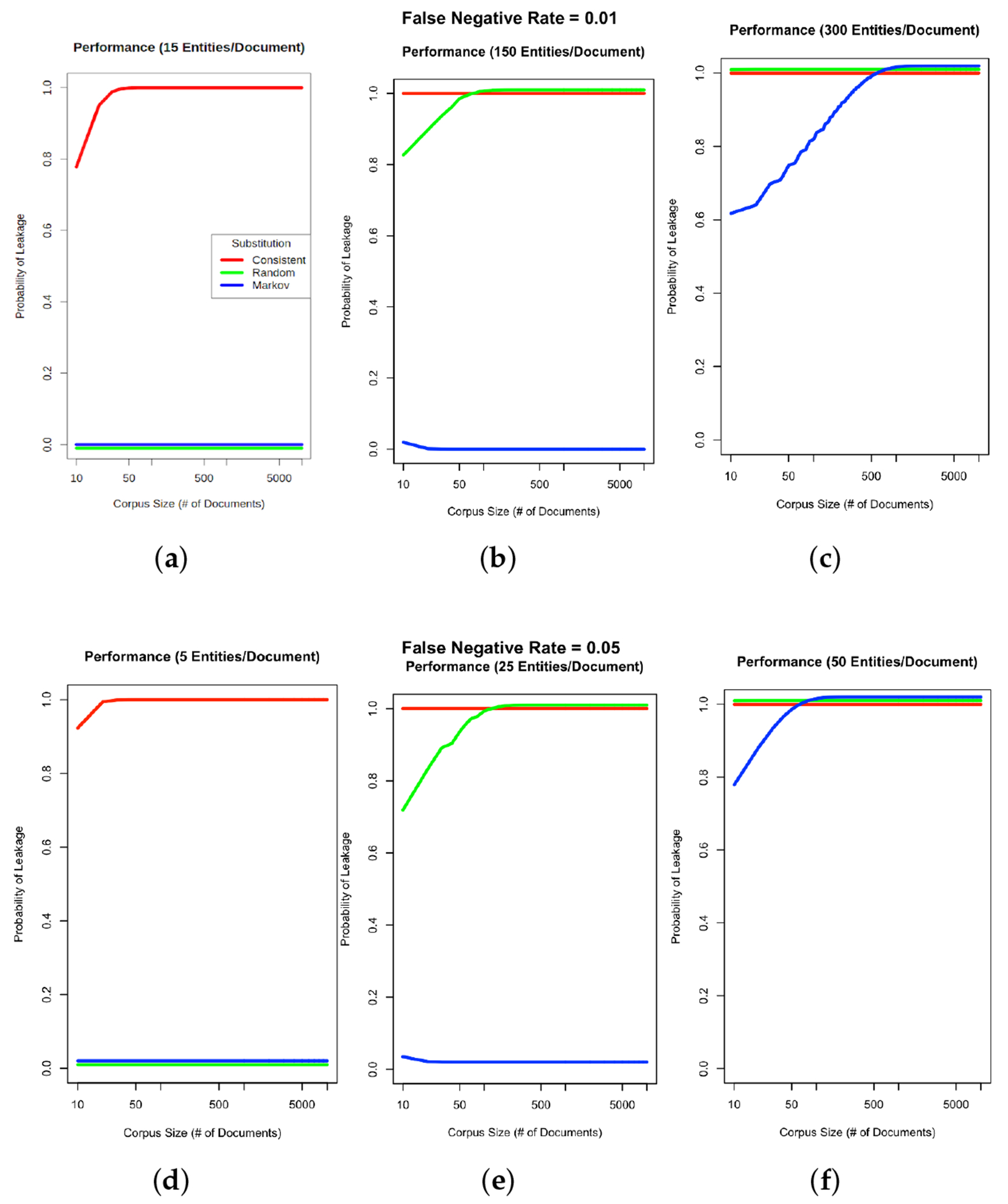
PHI leakage on synthetic corpora. Charts (**a**–**c**) show the probability of PHI leaking given a 1% PHI FNER and varying input corpus sizes with either 15, 150, or 300 entities (critical or otherwise) per document. Charts (**d**–**f**) assume a higher FNER of 5% with 5, 25, or 50 entities per document. Although leakage results are reported at the document level, patient level calculations would be the almost same but would use a pooled entity count given that multiple documents from the same patient would be aggregated. Red, green and blue lines indicate *Consistent*, *Random* and *Markov* substitution respectively.

Figure 4 shows the *Markov* strategy shows a lower document leakage rate on the UAB corpus. At a lower FNER (1% or less) its impact is more pronounced, with leakage eliminated at the lowest error rate. Even at a 5% error rate the strategy still performs substantially better than the two other strategies, which leak PHI close to 100% of the time. Figure 5 compares PHI leakage between the UAB and MIMIC corpus at the document level for discharge summaries only and indicates a similar substitution strategy performance between the two corpora, but dramatically lowers PHI leakage rates for the MIMIC corpus which contains significantly fewer critical PHI entities per discharge summary relative to UAB. The *Markov* substitution also has the highest performance on the synthetic corpora in Figures 6 and 7.

### 3.2. Surrogate Replacement Strategy: Effect on Information Extractions Tasks

Our results on the OUD dataset (Table 7) indicate that for context sensitive information extraction tasks, an encoder model fine-tuned on the target data set is not impacted by the surrogate substitution strategy. This is also true for most other information extraction tasks (Table 8), including entity extraction, even when the entity extraction model is not fine-tuned for the target dataset as is the case for MedSpacy. However, MedSpacy rule-based context prediction was impacted (1st row of Table 8) as was the SVM-based Averbis genomic marker pipeline as shown in Table 9. However, all MedSpacy divergences were <10^−4^ bits, confirming negligible distributional change. A sample of errors for MedSpacy POS-tagging and dependency parsing are shown in Tables 10 and 11 respectively. In general, the differences between the output of different surrogate substitution strategies did not correspond to our human intuition of the expected error types, as conceptually insignificant changes in numbers for ages and dates could result in unanticipated changes in the POS tag or the dependency class.

## 4. Discussion

### 4.1. PHI Leakage Replacement Strategy Evaluation

Our results indicate that a *Markov* replacement strategy can significantly reduce the chance of PHI leakage relative to a corpus released without substitution or using a traditional *Consistent* substitution strategy. This effect is seen in simulated PHI distributions (Figure 7) as well as real data from the UAB Discharge and MIMIC corpora (Figure 5), with the effect much more pronounced in the MIMIC corpora, which has almost no leakage at lower error rates. This discrepancy is due to the lower number of critical PHI entities contained in MIMIC corpus versus UAB Discharge. Since the release of our BRATsynthetic tool [46] and preprint [47], Simancek and Vydiswaran [48] have shown the effectiveness of a Markov chain-based surrogate replacement strategy using a Longformer BERT model for clinical text de-identification on a set of 400 discharge summaries. In this real-world data set, the average name leakage rate decreased from 13.1% with random replacement to 3.8% with *Markov* replacement of surrogates. In both real-world and simulated data the *Markov* substitution strategy is effective because it adds a variable amount of noise to a document data set (see Figure 6), at levels exceeding any original PHI signal that may not have been removed by the de-identification process. This shape of the *Markov* substitution strategy curve in Figure 5 is controlled by the state transition rate, which determines how often a surrogate is repeated. This could be adjusted in future work to reflect the expected number of FN errors in any entity to better conceal PHI leakage.

### 4.2. Larger Corpus Size

Our work suggests that the Markov substitution strategy allows for a larger release corpus size with the same or lower PHI leakage risk relative to *Consistent* or *Random* substitution strategies. This is true for a range of PHI entity counts and FNERs, as shown in Figure 7. Our results in Figures 4–6 focus on critical PHI, but the protective effect shown in Figure 7 applies to any PHI entity. We evaluated critical PHI because of the difficulty in assessing the impact or liability of inadvertent non-critical PHI release, similar to other work that focused on names only [48].

### 4.3. HIPS Strategy vs NER for PHI Protection

Our results in Figure 4 for real-world data and Figure 7 for simulated data show that the implementation of an effective surrogate replacement strategy using *Markov* models can have a greater impact on patient privacy protection than marginal gains in PHI NER performance provided by additional training data. For example, Dernoncourt et al. [4] show increased performance for artificial neural network and conditional random field de-identification algorithms with additional training data, but the total improvement as measured by F1 score shows that the maximum improvement difference was only about 2% between using 5% of available training data versus using all available training data for the MIMIC dataset. Our results indicate that the surrogate replacement strategy can have a greater impact in protecting PHI compared to even a 4% improvement in recall (from 5% to a 1%) in PHI NER software, depending on the total amount and distribution of PHI.

### 4.4. Impact on Information Extraction Tasks

Our results in Section 3.2 show that common information extraction tasks are not significantly impaired by the use of non-traditional PHI substitution strategies such as *Markov* or *Random* substitutions. The only clear exception to this is the MedSpacy “Context” task that includes the determination of the subject of a medical condition, something which *Random* or *Markov* replacement could interfere with. However, this was not replicated with the fine-tuned BioBERT used to generate results for Table 7, suggesting that this result may be more a limitation of the MedSpacy model.

### 4.5. Comparison to LLM-Based De-Identification Methods

LLM-based de-identification methods can produce contextually natural text, often exceeding template-driven systems in generation quality. However, they are computationally intensive, more expensive to operate and raise concerns about auditability and potential privacy leakage if safeguards are not rigorously applied. BRATsynthetic, on the contrary, is an inexpensive, efficient, and transparent deterministic surrogate substitution without specialized hardware, although at the cost of a more constrained narrative variety. In practice, these approaches should be viewed as complementary, with LLMs excelling in realism and adaptability, while BRATsynthetic offers practical scalability and predictable control, and its relative weakness in creating smooth transitions is less problematic in clinical documentation, where narratives are typically fragmented.

### 4.6. Limitations

BRATsynthetic uncouples surrogate generation from PHI NER and can thus leverage any independent improvements in PHI NER. The tradeoff is that PHI NER software is best positioned to recognize an appropriate substitution (such as one that maintains non-standard date formatting or even typos) whereas BRATsynthetic relies on PHI categories or its own regular expression-based interpretation of the text (if available) to generate the surrogate.

In assessing the impact of non-traditional replacement strategies for re-use, we sample only a limited set of task and datasets. However, we selected a clinical modifier task particularly well suited for the evaluation of surrogate changes, since it incorporates modifiers for Subject and DocRelTime whose mentions would be modified heavily in a *Random* or *Markov* corpus.

Both the faker library and BRATsynthetic are open source code, which could potentially assist an attacker since some synthetic surrogates are generated from lists including patient and location names. This vulnerability will be mitigated by private, user-provided pools of PHI for use as replacement values in an updated version of BRATsynthetic. Other forms of surrogate values, such as dates and randomly generated identifiers consisting of alphanumeric text, do not have this vulnerability. Finally, we have not performed a complexity or runtime analysis on the faker library, but scaling on large datasets is feasible, as shown in Table 6.

## 5. Conclusions

We show that a Markov substitution strategy can reduce PHI leakage on two real world corpora and on synthetic PHI distributions using a range of realistic false negative PHI NER rates and entity distributions and make our software freely available. Although our work here shows the theoretical and statistical utility of the Markov-based surrogate substitution method, further evaluation of the software implementation on a wider range of benchmarks including human validation would be useful, as would an assessment against more complex re-identification attempts including parrot [25] and LLM-based attacks [26].

## Figures and Tables

**Table 1.** Comparison of de-identification tools by surrogate substitution strategy and open-source availability. Simple indicates placeholder-style substitution (e.g., [NAME]), Label indicates structured identifiers (e.g., [PATIENT_ID_001]), Consistent indicates that a given PHI entity is always replaced with the same realistic surrogate. Markov indicates the realistic surrogate replacement using a Markov-chain strategy described here in the Section 2. A ✓ indicates the presence of that feature and a ✘ indicates its absence.

Tool or Method	Surrogate Strategy				Open Source
	Simple	Label	Consistent	Markov	
Presidio [19]	✓	✘	✘	✘	✓
Deid [20]	✘	✓	✘	✘	✓
MIST v2 [14]	✘	✓	✘	✘	✓
CliniDeID [10]	✓	✘	✓	✘	✓
Stanford HIPS System [17]	✓	✘	✓	✘	✓
nference De-id [18]	✓	✘	✓	✘	✘
BRATsynthetic [21]	✓	✘	✓	✓	✓

**Table 2.** BRATsynthetic safe harbor element replacement.

HIPAA Safe Harbor Category	Type	Description	Critical PHI	BRATsynthetic Replacement	MIMIC Identifier Table
NAMES	DOCTOR	Health care provider name	No	DOCTOR	DOCTOR
	PATIENT	Patient name	Yes	PATIENT	PATIENT
	USERNAME	User IDS of provider	No	USERNAME	-
GEO LOCATION	LOC-OTHER	Identifiable locations and landmarks	No	LOCATION-OTHER	LOC-OTHER
	HOSPITAL	Hospital or clinic name	No	HOSPITAL	HOSPITAL
	WARD	Ward or unit name	No	-	WARD
	ZIP	Zip code	No	ZIP	ZIP
	ORGANIZATION	Employers	No	ORGANIZATION	ORGANIZATION
	COUNTRY	Country	No	COUNTRY	COUNTRY
	STATE	State or province name	No	STATE	STATE
	CITY	Name of city	No	CITY	LOC-OTHER
	STREET	Street address	No	STREET	STREET
DATES	DATE	Year	No	DATE (regex)	DATE
		Month/Day	No	DATE (regex)	DATE
		Day of the week	No	DATE (regex)	-
	HOLIDAY	Holidays	No	DATE (regex)	HOLIDAY
	AGE	AGE ≥ 90	No	AGE	AGE_90_ANDUP
		AGE < 90	No	AGE	-
PHONE	PHONE	Telephone numbers	Yes	PHONE	PHONE
		Vehicle			
VEHICLE IDS	VEHICLE_ID	identification number or license	Yes	IDNUM (regex)	IDNUM
FAX	FAX	Fax numbers	Yes	PHONE	PHONE
DEVICE IDS	DEVICE IDS	Device identifiers and serial numbers	Yes	DEVICE (regex alphanumeric)	IDNUM
IDNUM	IDNUM	License and health plan numbers	Yes	IDNUM (regex)	-
MEDICAL RECORD	MEDICAL RECORD	Medical record number	Yes	IDNUM (alphanumeric)	-
SSN	SSN	Social security number	Yes	IDNUM (regex)	SSN
ACCOUNT ID	ACCOUNT ID	Account numbers	Yes	ACCOUNT ID	
EMAIL	EMAIL	Email address	Yes	EMAIL	-
URL	URL	URL	No	URL	-
BIOMETRIC ID	BIOID	Biometric identifiers, including finger and voice prints	NA	BIOID (alphanumeric)	-
IP ADDRESS	IP ADDRESS	Internet Protocol Address	No	URL	-

**Table 3.** Differences between safe harbor elements and BRATsynthetic.

Safe Harbor Element	Brat Synthetic Category
IMAGE	Images are not de-identified
UNIQUE	Unique identifying phrases must be manually redacted
Not covered under safe harbor	PROFESSION (also a category in i2b2)
Not covered under safe harbor	TIME (exclusive to BRATsynthetic)

**Table 4.** Substitution strategies in BRATsynthetic. All are HIPS strategies except “Simple”. MSRS refers to the “Maximum Surrogate Repeat Size” and NA means Not Applicable.

	Substitution Strategy			
	Simple	Consistent	Random	Markov
Original Name	Sandy	Sandy	Sandy	Sandy
1st Surrogate Replacement	*ENTITY_NAME*	Sara	Kim	Sara
2nd Surrogate Replacement	*ENTITY_NAME*	Sara	Nisha	Sara
3rd Surrogate Replacement	*ENTITY_NAME*	Sara	Cathy	Ann
4th Surrogate Replacement	*ENTITY_NAME*	Sara	Maria	Maria
5th Surrogate Replacement	*ENTITY_NAME*	Sara	Hannah	Maria
6th Surrogate Replacement	*ENTITY_NAME*	Sara	Lin	Maria

PHI Similarity to Original	Lowest	High	Low	Intermediate
MSRS	NA	NA	1	3

**Table 5.** Critical entity distribution by corpus.

Corpus	UAB Total	UAB Discharge	MIMIC Discharge	UAB Total
Type	Document	Document	Document	Patient
Mean	388.5	355.6	6.8	8123.0
Median	224	199	5	985
Range	2–2545	10–2414	2–76	7–321,945

**Table 6.** BRATsynthetic Runtime Statistics.

Machine	Documents	Words	PHI Entities	Runtime *
3.4 GHz Quad-Core Intel i7 CPU with 32 GB 1600 MHz DDR 3	28,547	32,432,577	1,710,386	20.9 s system time

* Note: Average of five (5) runs.

**Table 7.** Clinical modifier extraction macro F1 score on the OUD dataset with a fine-tuned BioBERT pipeline. NER results are reported based on partial matches. None indicates identified text.

Task	Traditional		HIPS			*p*-Value

	None	Simple	Consistent	Random	Markov	
NER	0.723	0.730	0.723	0.720	0.722	0.704

Subject	0.914	0.936	0.918	0.926	0.927	0.868
DocTime	0.932	0.926	0.925	0.934	0.925	0.105
Negation	0.972	0.969	0.975	0.975	0.972	0.469

**Table 8.** HIPS strategy Jensen–Shannon divergence [bits] on the OUD dataset using standard MedSpacy. We provided the Entity identification framework with a custom set of OUD-specific rules.

MedSpacy Task	Consistent	Random	Markov
Context	7.9140 × 10^−7^	3.90545 × 10^−6^	1.3125 × 10^−5^
Dependency Parse	6.0994 × 10^−5^	6.7103 × 10^−5^	6.0274 × 10^−5^
Tokenization	4.9393 × 10^−2^	4.8248 × 10^−2^	4.8471 × 10^−2^
Entity Extraction	9.1735 × 10^−2^	9.1149 × 10^−2^	9.1441 × 10^−2^
POS Tagging	4.5877 × 10^−5^	4.3207 × 10^−5^	4.2839 × 10^−5^
Spans	1.6906 × 10^−4^	1.6906 × 10^−4^	1.6906 × 10^−4^

**Table 9.** Averbis genomic marker pipeline genomic marker counts and error analysis for five OUD documents where results differed from the original data. A red text color indicates any hypothesized surrogate-induced error we could identify. Note that the MRN 1324616 shown in the Consult Note is synthetic and not a real patient MRN. Abbreviations for HIPS substitution strategies are *Consistent* (Cons), *Random* (Rand), and *Markov* (Mark).

Document Type	HIPS Substitution Strategy				Explanation
	None	Cons	Rand	Mark	
Psychiatry Note	0	1	0	0	ER: UAB ER, -> ER: ACH ER
MR Breast Diagnostic Bil wo+w contrast	2	2	1	2	Estrogen receptor positive status [ER+]
Emergency Department Note	0	0	0	2	recently seen in ER on 4/4
Emergency Department Note	0	1	2	2	Pt was recently seen in ER on 02/08
Consult Note	15	15	15	16	MRN: 1324616 Progesterone receptor

**Table 10.** Universal Part of Speech tagset [45] comparison of two examples mentioned, bold text indicates for that portion of the mention text indicates the dependencies for that text are shared by all substitution methods. POS tags are color-coded: PROPN, NOUN, NUM.

Type	Example Mentions				
Consistent	36 Years	Ethanol level	07/17/19 08:48	Ampheta	07/17/19
Markov	41 Years		07/14/19 23:59		07/12/19
Random	37 Years		07/13/19 22:17		07/17/19
Simple	[AGE][AGE]		[DATE][TIME]		[DATE]

**Table 11.** Comparison between incoming dependencies for 2 example mentions, bold text indicates for that portion of the mention text indicates the dependencies for that text are shared by all substitution methods. Dependencies are color-coded: CONJ, APPOS, NUMMOD, Compound, PUNCT, ADVMOD, NPADVMOD, ROOT.

Type	Example Mentions			
Consistent	Date &	Time 07/13/2019 18:32	Ethanol	level 07/17/19 08:48
Markov		Time 07/13/2019 22:35		level 07/14/19 07:54
Random		Time 07/17/2019 06:44		level 07/13/19 22:17
Simple		Time [DATE][TIME]		level [DATE] [TIME]

## Data Availability

The deidentified Delirium and Opiate Use Disorder (OUD) dataset will be made available to interested parties who wish to replicate results with a Data Use Agreement (DUA) prohibiting distribution and re-identification attempts.
